# HMGA2 promotes cancer metastasis by regulating epithelial–mesenchymal transition

**DOI:** 10.3389/fonc.2024.1320887

**Published:** 2024-02-01

**Authors:** Qing Ma, Sisi Ye, Hong Liu, Yu Zhao, Yan Mao, Wei Zhang

**Affiliations:** ^1^ General Practice Ward/International Medical Center Ward, General Practice Medical Center, West China Hospital, Sichuan University/West China School of Nursing, Sichuan University, Chengdu, China; ^2^ Emergency Department of West China Hospital, Sichuan University/West China School of Nursing, Sichuan University, Chengdu, China

**Keywords:** HMGA2, epithelial-mesenchymal transition, extracellular matrix, cancer, gene therapy

## Abstract

Epithelial–mesenchymal transition (EMT) is a complex physiological process that transforms polarized epithelial cells into moving mesenchymal cells. Dysfunction of EMT promotes the invasion and metastasis of cancer. The architectural transcription factor high mobility group AT-hook 2 (HMGA2) is highly overexpressed in various types of cancer (e.g., colorectal cancer, liver cancer, breast cancer, uterine leiomyomas) and significantly correlated with poor survival rates. Evidence indicated that HMGA2 overexpression markedly decreased the expression of epithelial marker E-cadherin (CDH1) and increased that of vimentin (VIM), Snail, N-cadherin (CDH2), and zinc finger E-box binding homeobox 1 (ZEB1) by targeting the transforming growth factor beta/SMAD (TGFβ/SMAD), mitogen-activated protein kinase (MAPK), and WNT/beta-catenin (WNT/β-catenin) signaling pathways. Furthermore, a new class of non-coding RNAs (miRNAs, circular RNAs, and long non-coding RNAs) plays an essential role in the process of HMGA2-induced metastasis and invasion of cancer by accelerating the EMT process. In this review, we discuss alterations in the expression of HMGA2 in various types of cancer. Furthermore, we highlight the role of HMGA2-induced EMT in promoting tumor growth, migration, and invasion. More importantly, we discuss extensively the mechanism through which HMGA2 regulates the EMT process and invasion in most cancers, including signaling pathways and the interacting RNA signaling axis. Thus, the elucidation of molecular mechanisms that underlie the effects of HMGA2 on cancer invasion and patient survival by mediating EMT may offer new therapeutic methods for preventing cancer progression.

## 1 Introduction

The extracellular matrix (ECM) is a non-cellular support structure that exists in all tissues, and each organ has a unique ECM composition (1). Apart from a support structure for tissue architecture, the ECM is actually a dynamic compartment that modulates and regulates cell functions, such as adhesion, migration, proliferation, and differentiation (2, 3). It is composed of numerous matrix macromolecules, including collagen, laminin, elastin, fibronectin, and hyaluronic acid (4, 5). Epithelial–mesenchymal transition (EMT) is an important physiological process through which polarized epithelial cells are transformed into moving mesenchymal cells (6). The main phenotypic changes in EMT are the downregulation of E-cadherin (CDH1) and the upregulation of vimentin (VIM) (7). EMT is active during embryonic development, invasion, metastasis, and therapeutic resistance in cancer (6).

The progression of EMT is influenced by the expression of multiple transcription factors, such as high mobility group AT-hook 2 (HMGA2) (8, 9). The HMGA proteins act as non-histone components of chromatin (10) and participate in regulating chromatin structure and DNA recombination (11, 12). The HMGA family consists of two members, HMGA1 and HMGA2, which play important roles in several processes (e.g., gene regulation, cell cycle changes) (13, 14). HMGA2 is relatively abundant in the early embryo and most types of cancer; however, it exhibits lower expression in adult tissues (15, 16). Research has shown overexpression of HMGA2 in a variety of cancers [e.g., colorectal cancer (CRC), liver cancer, breast cancer (BC), and uterine leiomyomas], suggesting an essential role in tumor development and invasion (17–20). Furthermore, clinical studies have revealed a significant correlation between the expression of HMGA2 in tissue samples and the grading and metastasis of cancer, as well as the survival rate of patients with cancer (21, 22). Wu et al. demonstrated that high levels of HMGA2 were significantly correlated with poor survival of patients with BC, particularly those with stage II–III disease. In addition, gene set enrichment analysis indicated that HMGA2 expression was positively correlated with gene expression of the mesenchymal phenotype (23). HMGA2 expression is obviously increased in BC, and interference with HMGA2 can inhibit the metastasis and invasion of tumors (24). Moreover, the expression levels of EMT-related proteins were decreased after interfering with HMGA2 expression (24). Similarly, another study has shown that HMGA2 induced metastasis of human epithelial cancers by activating the expression of transforming growth factor beta type II receptor (TGFβRII) (8). Activation of the EMT process was regarded as a major driver of aggravation from tumorigenesis to metastasis (25). Moreover, an *in-vitro* study strongly suggested a key role of HMGA2 in EMT (26, 27). Most previous studies demonstrated that HMGA2 plays a key role in cancer growth, invasion, and the EMT phenotype, which involved the interactions of multiple signaling proteins and non-coding RNAs (28, 29).

Therefore, targeting HMGA2 for the regulation of EMT may be an important strategy for combating tumor metastasis, recurrence, and drug resistance. In this literature review, we highlight the EMT-induced role of HMGA2 in tumor development and invasion and discuss signaling pathways that may be affected by HMGA2. According to available evidence, HMGA2 suppression may be a promising target for cancer therapy.

## 2 Physiological function of HMGA2

**Figure 1:**
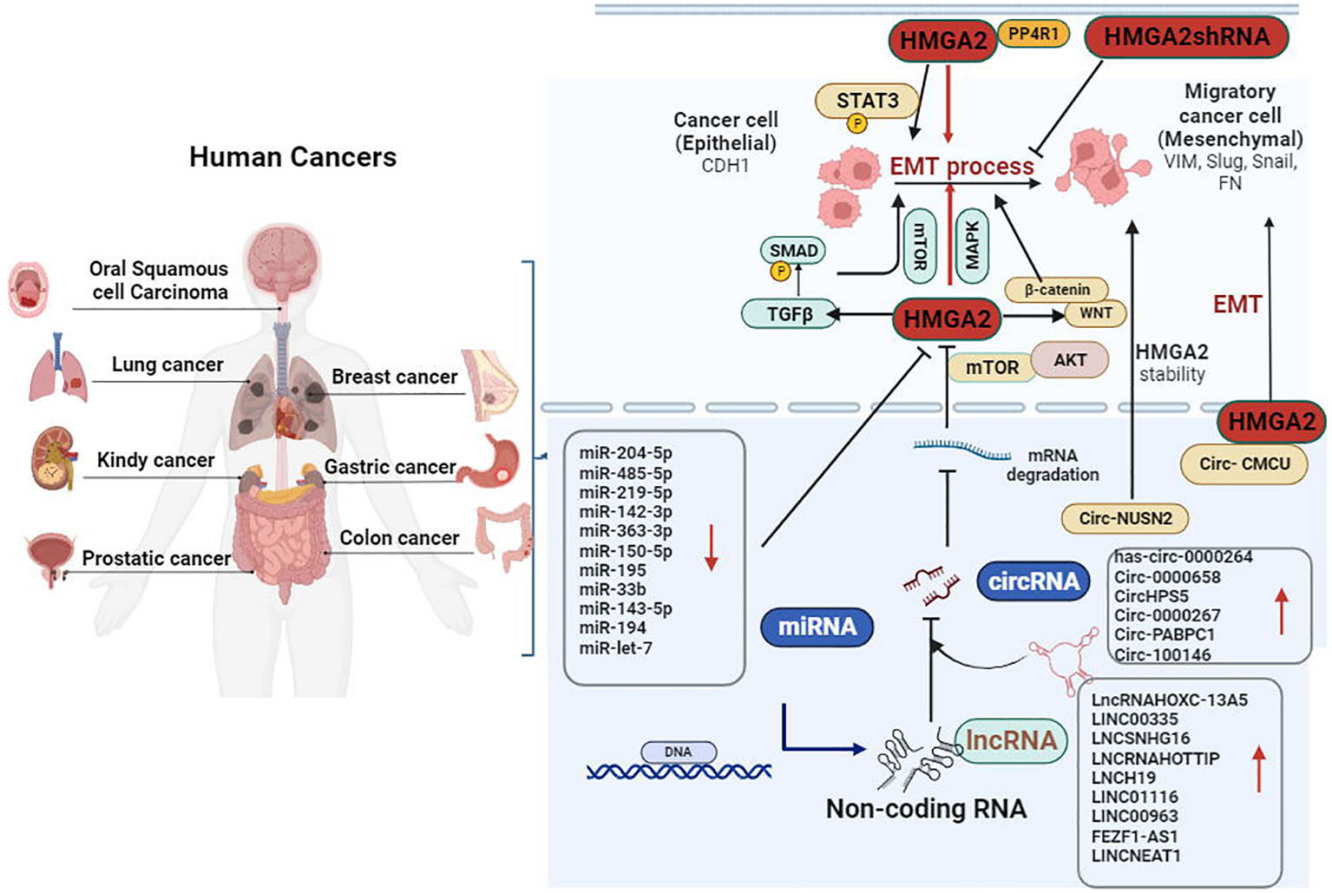
Dysfunction of epithelial–mesenchymal transition (EMT) is an important factor promoting the invasion and metastasis of cancer. HMGA2 overexpression decreased the expression of epithelial marker CDH1, whereas it increased the levels of VIM, Snail, Slug, and FN by targeting the TGFβ/SMAD, MAPK, and WNT/β-catenin signaling pathways. Furthermore, new classes of non-coding RNAs (miRNAs, circRNAs, and lncRNAs) play an essential role in the process of HMGA2-induced metastasis and invasion of cancer by accelerating the EMT process. CDH1, cadherin 1; circRNAs, circular RNAs; β-catenin, beta-catenin; FN, fibronectin; HMGA2, high mobility group AT-hook 2; lncRNAs, long non-coding RNAs; MAPK, mitogen-activated protein kinase; TGFβ, transforming growth factor beta; VIM, vimentin.

The HMGA family includes four subtypes, namely, HMGA1a, HMGA1b, HMGA1c, and HMGA2 (30, 31). Among them, HMGA1a–c are different splicing products of the HMGA1 gene, while HMGA2 is a gene product of HMGA2 (32). As a member of the HMGA family, HMGA2 comprises five exons. The first three exons contain DNA-binding domains (termed AT-hook motifs) (15, 33). The “AT-hook” DNA-binding motif contains a unique palindrome sequence PGRGP, surrounded by one or two positively charged amino acids (i.e., lysine or arginine) on each side (34, 35). This special structure facilitates the binding of HMGA2 to the AT-rich regions and an acidic C-terminal tail in the small grooves of DNA. This leads to ordered changes in DNA structure and further affects several processes (e.g., changes in chromatin structure, DNA damage/repair, DNA replication and transcription) (36–38). It is also possible to activate the transcription of target genes by competing with the junction histone H1 to open dense chromatin (39). HMGA2 is highly expressed in the early developmental stage and participates in the differentiation of mesenchymal stem cells during fetal development (40). However, it remains silent in normal mature tissues, except for lung tissue, kidney tissue, and synovial tissue (41, 42). The loss of epithelial markers and the acquisition of mesenchymal markers are typical characteristics of EMT, which play a key role in embryonic development (43). It was reported that high expression of HMGA2 in cancer changes the cell phenotype from epithelial to mesenchymal (44). In non-small cell lung cancer (NSCLC), the protein phosphatase 4 regulatory subunit 1 (PPP4R1) interacts with HMGA2 to promote cell migration and metastasis via activating EMT (45). Furthermore, Kou et al. suggested that HMGA2 facilitated metastasis and the EMT process in renal cell carcinoma cells by the TGFβ/SMAD2 pathway (46). In prostate cancer, AMPK plays a critical role in the promotive effect of HMGA2 on EMT (47). Similarly, another study revealed that HMGA2 was a direct regulatory target for various EMT-related non-coding RNAs (43). Therefore, HMGA2 may be essential in regulating the EMT process in cancer (**Figure 1**).

## 3 Regulatory role of the HMGA2 axis in the EMT process in cancer

### 3.1 Gastric cancer

EMT is a key factor in the invasion and metastasis of gastric cancer (GC) (48). It was demonstrated that the transcription factors zinc finger E-box-binding homeobox 1 (ZEB1) and Snail induced EMT by suppressing the expression of CDH1 (49–51). Analysis of surgical specimens of GC and clinical pathological data from patients with cancer showed that HMGA2 overexpression was compared with normal epithelium (52). However, HMGA2 knockdown obviously increased the expression of CDH1 and decreased that of N-cadherin (CDH2), ZEB1, and Snail (52). Moreover, similar research found that HMGA2 decreased the expression levels of Snail and β-catenin in GC cells, indicating that HMGA2 may promote the migratory capacity of GC cells by regulating EMT (53). The key role of the WNT/β-catenin pathway in regulating cell adhesion and migration is closely related to EMT (54, 55). Zha et al. demonstrated that HMGA2 induced the EMT phenotype by targeting the WNT/β-catenin pathway in MKN45 cells (56) (**Figure 1**). Dysfunction of non-coding RNAs has been associated with the development of malignant tumors (57, 58). Accumulating evidence indicates that HMGA2 primarily acts as a downstream factor of long non-coding RNAs (lncRNAs) for regulating tumor progression (59, 60). Overexpression of circ_0000267 in GC tissues and cell lines is related to cancer progression through regulation of the miR-503-5p/HMGA2 axis (61). In GC stem cells, it was demonstrated that knockdown of FEZF1 antisense RNA 1 (FEZF1-AS1) suppressed GC stem cell progression. Notably, FEZF1-AS1 promoted EMT, invasion, and migration of GC stem cells via the miR-363-3p/HMGA2 pathway (62). Collectively, these results indicated that HMGA2 may be a key target for inhibiting EMT in GC progression.

### 3.2 Lung cancer

The functions of HMGA2 in lung cancer have been studied extensively (63, 64). For instance, HMGA2 promoted proliferation, apoptosis, and EMT in lung cancer cells and was a biomarker for lung adenocarcinoma (64). Li et al. presented evidence indicating that the loss of three transcription factors (i.e., Foxa2, Cdx2, and Nkx2-1) is sufficient to induce the upregulation of Tks5long, HMGA2, and the EMT mediator Snail (65). In addition, PPP4R1 cooperated with HMGA2 to promote EMT by activating the mitogen-activated protein kinase/extracellular signal-regulated kinase (MAPK/ERK) signaling pathway, thereby accelerating migration and invasion in NSCLC (66). The interaction between miRNAs and HMGA2 to promote EMT in lung cancer has been demonstrated. It was shown that miR-195 could target and downregulate HMGA2 to induce EMT and proliferation in lung cancer cells (43). As a negative regulator of tumors, fragile histidine triad diadenosine triphosphatase (FHIT) can inhibit metastasis in lung cancer (67). Overexpression of FHIT leads to the downregulation of the EMT-related genes VIM and fibronectin. This effect is exerted by activating miR-30c/HMGA2 to decrease HMGA2 (67). In addition, miR-150-5p expression was significantly suppressed in cancer stem cells, and this observation may be related to disease progression and poor survival in patients with lung cancer. Overexpression of miR-150-5p significantly suppressed the metastasis of NSCLC cells by directly targeting HMGA2 signaling (68). Furthermore, circular RNAs (circRNAs) also play an important regulatory role in the miRNA/HMGA2 pathway. For example, interference of circ_100565 is in favor of NSCLC cell progression by targeting the miR-506-3p/HMGA2 axis (69).

### 3.3 Colorectal cancer

Most previous studies in CRC demonstrated that HMGA2 contributed to disease progression and was associated with poor patient survival (20, 28, 70). EMT is a key biological process in the progression and invasion of CRC cells (71–73). Research demonstrated that HMGA2 was regulated by directly binding to the fibronectin 1 (FN1) and interleukin 11 (IL11) promoters. This process accelerates EMT in CRC cells via the phosphorylated signal transducer and activator of transcription 3-dependent (STAT3-dependent) pathway (28, 74). In addition, HMGA2 short-hairpin RNA (shRNA) attenuated the proliferation and invasion, while exogenous HMGA2 expression induced an increase in epithelial markers and a reduction in mesenchymal markers. These effects were achieved by binding to the regulatory area of Slug (75). Notably, HMGA2 plays an essential role in the regulation of the EMT phenotype in CRC, which is associated with miRNA regulation (75, 76). In CRC, highly expressed long intergenic non-protein coding RNA 963 (LINC00963) was related to poor prognosis. LINC00963 promoted the expression of EMT-related genes and invasion of CRC through the miR-532-3p/HMGA2 pathway (77). Acting as an inhibitory regulator of upstream genes, overexpression of miR-194 suppressed the expression of HMGA2, thereby improving the cell survival, EMT process, and drug resistance in CRC (78). Similarly, increased miR-330 levels also reduced the phosphorylation of AKT and STAT3 and downregulated the expression of SMAD3, Snail, and vascular endothelial growth factor A (VEGFA) by suppressing HMGA2 (44, 79). Moreover, it was demonstrated that tumor protein p53-induced (TP53-induced) miR-1249 suppressed the disease progression and angiogenesis by regulating the VEGFA-mediated AKT/mechanistic target of rapamycin kinase (AKT/mTOR) pathway. This observation further supported that TP53-induced miR-1249 inhibited the EMT in CRC by targeting VEGFA and HMGA2 (80). Numerous studies reported that dysregulated expression of circRNAs participates in the pathological processes of CRC (26, 45, 81). NOP2/Sun RNA methyltransferase 2 (NSUN2) is an N6-methyladenosine-modified circRNA highly expressed in CRC cells and patients. It has been shown to enhance the stability of HMGA2, increase CDH1 expression, and decrease VIM expression. These findings suggested that circRNA NSUN2 accelerates the EMT process in CRC cells by targeting the HMGA2 pathway (82). Additionally, circRNA 100146 was highly expressed in CRC patients and cells. Knockdown of circRNA 100146 in CRC cells disrupted the proliferation and EMT by sponging the miR-149/HMGA2 pathway (33257506). The circRNA in exosomes participated in intercellular communication and may be closely related to tumor metastasis (83). It was found that exosomal circRNA poly(A) binding protein cytoplasmic 1 (PABPC1) promoted the EMT-mediated CRC liver metastasis by increasing the expression of HMGA2 and bone morphogenetic protein 4/ADAM metallopeptidase domain (19 BMP4/ADAM19) (84). To some extent, these research studies revealed the role of HMGA2 and the potentially involved mechanism in CRC.

### 3.4 Breast cancer

BC remains the main cause of cancer-related disease burden in women (85). EMT leads to the development of drug resistance in BC cells, and inhibition of the EMT process can improve drug sensitivity (86, 87). Accumulating evidence has shown that HMGA2 is highly expressed in patients with BC. This high expression was positively related to advanced tumor grade. Moreover, HMGA2 enhanced the migratory and invasive abilities of BC cells by inducing EMT (23, 88, 89). Multiple signal proteins play essential roles in the process of HMGA2, participating in the metastasis and invasion of BC by accelerating EMT. Kolliopoulos et al. investigated HMGA2-depleted cells, which were stimulated with TGFβ. Genes known to be upregulated during EMT, such as Snail, serpin family E member 1 (SERPINE1), and FN1, were suppressed. This evidence reinforced the key role of HMGA2 in TGFβ-induced EMT (38). In addition, the WNT10B network β-catenin/HMGA2/enhancer of zeste 2 polycomb repressive complex 2 subunit (β-catenin/HMGA2/EZH2) signaling was related to survival and metastasis in triple-negative BC (90). Similarly, in triple-negative BC, HMGA2 suppressed the ubiquitination of Yes-associated protein (YAP) and modulated YAP stability, thereby regulating the EMT in tumors (24). Furthermore, the knockdown of combined HMGA2 and BTB domain and CNC homolog 1 (BACH1) obviously decreased cell migration and EMT. These results suggested that combined targeting of HMGA2 and BACH1 is an effective therapeutic strategy for treating BC (91). It has been indicated that cancer stem cells may arise from non-stem cancer cells upon microenvironment signals (92). A study demonstrated that the Lin-28B/let-7/HMGA2 axis was activated by STAT3/nuclear factor kappa B (STAT3/NFKB) to regulate the EMT/cancer stem cell formation; of note, HMGA2 plays a major role in this axis (93). Recently, the differential expression of clinical pathological factor-related miRNAs has been associated with HMGA2-induced EMT. For instance, miR-33b was lowly expressed in BC tissues and suppressed the EMT progress and invasion of BC despite targeting HMGA2, spalt-like transcription factor 4 (SALL4), and twist family bHLH transcription factor 1 (TWIST1) (94). Furthermore, acting as a negative regulatory factor, miR-143-5p could decrease VIM and CDH2 protein expression and increase CDH1 protein expression by directly targeting HMGA2 (95). Research that focused on the effects of modifications on miRNA has demonstrated that N6-methyladenosine modification of RNAs is crucial for cancer progression (96). Zhao et al. reported that methyltransferase 3, N6-adenosine-methyltransferase complex catalytic subunit (METTL3) regulated EMT in BC by targeting the metastasis-associated lung adenocarcinoma transcript 1/miR-26b/HMGA2 (MALAT1/miR-26b/HMGA2) pathway. This finding may provide an array of new therapeutic targets, including HMGA2, for the treatment of BC (97). In addition, new classes of non-coding RNAs (circRNAs and lncRNAs) play an essential role in the process of HMGA2-induced metastasis and invasion of BC by accelerating the EMT process. In BC, the upregulated expression of circHMCU favored disease progression; circHMCU promotes cell proliferation and metastasis by binding to HMGA2 (98). As an example of regulation of the lncRNA/HMGA2 pathway, the lncRNA nuclear paraspeckle assembly transcript 1 (NEAT1) that targeted the miR-211/HMGA2 axis can contribute to the EMT phenotype, thus promoting BC metastasis and chemoresistance (99).

### 3.5 Esophageal cancer

In esophageal cancer, the high expression of HMGA2 plays an essential role in regulating EMT (100). Esophageal squamous cell carcinoma (ESCC) is currently the most common type of esophageal tumors. Currently, research is mainly focused on non-coding RNAs as the molecular mechanisms related to HMGA2 during EMT in esophageal cancer (100). Mechanistic analysis revealed that miR-490-3p is bound to the 3′-untranslated region of HMGA2 to downregulate its expression. This effect suppressed the invasion, migration, and EMT of ESCC cells (101). It was elucidated that HMGA2 functions as an oncogene; silencing HMGA2 decreased the expression levels of CDH2, VIM, and Snail in ESCC cells by negatively regulating miR-204-5p (72). However, miR-125b-5p acts as an upstream target; its overexpression suppresses cell invasion by decreasing HMGA2 in ESCC (102). In addition, upregulation of hsa_circ_0006948 can promote the proliferation, migration, and invasion of ESCC cells. These effects are exerted by sponging miRNA-490-3p to increase HMGA2 expression (103).

### 3.6 Other types of cancer

An increasing body of evidence has shown that HMGA2 is overexpressed in most tumor tissues or cancers, except for GC, NSCLC, CRC, BC, and esophageal cancer, also including thyroid cancer, bladder cancer, endometrial cancer, cervical cancer, tongue cancer, and kidney cancer (104–109) (**Table 1**). High expression of HMGA2 was associated with EMT and metastasis and predicted poor prognosis in patients with cancer (104–106, 125, 126). Recent research showed that exosomal HMGA2 from the Epstein–Barr virus promoted tumor metastasis and EMT (109). Data demonstrated that HMGA2 can regulate the TGFβ/SMAD and MAPK signaling pathways to induce tumor cell invasion and migration. For example, knockdown of HMGA2 significantly inhibited EMT in nasopharyngeal carcinoma cell lines by targeting the TGFβ/SMAD3 signaling pathway (105). In addition, in renal cell carcinoma cells *in vitro*, overexpression of HMGA2 facilitated the EMT process through the TGFβ/SMAD2 signaling pathway (46). Similarly, activation of the TGFβ signaling pathway may be a key step in EMT, induced by HMGA2 in human epithelial cancers (8). Furthermore, the upregulation of HMGA2 induced EMT phenotypes through regulation of the MAPK pathway (47). Transcription regulatory factors also promoted the transfer and expression of HMGA2, thereby promoting the growth and metastasis of ovarian cancer (110). Massive miRNAs have been clarified to affect EMT and invasion of cancers through regulating the expression of HMGA2. In endometrial cancer, cell proliferation and the EMT were promoted after HMGA2 overexpression which resulted from miR-302a-5p/367-3p downregulation (107). Furthermore, miR-302a-5p/367-3p and miR-142-3p act as tumor-suppressive miRNAs, playing a key role in the regulation of EMT by targeting HMGA2 in human cervical cancer (117). In addition, miR-219-5p and miR-154 suppressed the growth and EMT of prostate cancer cells by directly sponging the expression of HMGA2 (114, 115). In laryngeal squamous cell carcinoma, HMGA2 expression was negatively related to the levels of miR-98, and the miR-98/HMGA2/periostin (miR-98/HMGA2/POSTN) axis played an important role in reversing EMT (112). Furthermore, it has been reported that miR-101, miR-204-5p, miR-485-5p, and miR-150 reverse metastasis and EMT by targeting the HMGA2 (29, 111, 113, 116, 118). Another research study demonstrated that miR-33b reversed the EF24-mediated suppression of EMT by suppressing HMGA2 expression in melanoma (127). It was found that lncRNAs significantly affect the EMT of cancers by modulating the miRNA/HMGA2 axis. In tongue squamous cell carcinoma cells, HOXA distal transcript antisense RNA (HOTTIP) knockdown suppressed the cell migration and EMT by the miR-124-3p/HMGA2 axis, and the H19/let-7a/HMGA2/EMT pathway was also involved in the regulation of EMT (123, 124). In addition, in nasopharyngeal carcinoma, HOXC13 antisense RNA (HOXC13-AS) promoted EMT-induced invasion via regulating the miR-383-3p/HMGA2 pathway (121). Similarly, modulation of the miR-424-5p/HMGA2 pathway by LINC01116 indicated a potential pathway for overcoming the resistance of osteosarcoma to chemotherapy (122). In addition, the small nucleolar RNA host gene 16/let-7b-5p/HMGA2 (SNHG16/let-7b-5p/HMGA2) axis and the lncRNA LINC00355/miR-424-5p/HMGA2 axis play an important role in the EMT of cancers. Suppression of these signaling axes can prevent tumor metastasis (69, 122, 128, 129). Apart from lncRNAs, circRNAs (as upstream regulatory targets) affect EMT in cancers by modulating the miRNA/HMGA2 axis. A study indicated that hsa_circ_0000264 may serve as a target for the treatment of head and neck squamous cell carcinoma-EMT by regulating the hsa-let-7b-5p/HMGA2 pathway (119). Studies showed that circ_0000658 was highly upregulated in bladder cancer; nevertheless, circ_0000658 knockdown reduced the EMT phenotypes by regulating the miR-498/HMGA2 pathway (120).

**Table 1:** HMGA2 promotes EMT in multiple types of cancer.

Cancer type	HMGA2 expression	Signaling network	Change in EMT	
			Upregulation	Downregulation
Nasopharyngeal carcinoma (105)	Upregulation	HMGA2/TGFβ/SMAD3	VIM, Snail	CDH1
Renal cell carcinoma (46)	Upregulation	HMGA2/TGFβ/SMAD2	CDH2, TWIST1, TWIST2	CDH1
Human epithelial cancers (8)	Upregulation	HMGA2/TGFβ	N/A	CDH1
Prostate cancer (47)	Upregulation	HMGA2/MAPK/ERK	Snail, TWIST1, VIM	N/A
Ovarian cancer (110)	Upregulation	BACH1/HMGA2	Snail, SNAI2	N/A
Endometrial cancer (107)	Downregulation	miR-302a-5p/367-3p/HMGA2	CDH1	Slug, Snail, CDH2
Pancreatic cancer (111)	Downregulation	miR-101/HMGA2	CDH1	VIM, CDH2
Laryngeal squamous cell carcinoma (112)	Downregulation	miR-98/HMGA2/POSTN	CDH1	Snail, ZEB1
Bladder cancer (113)	Downregulation	miR−485−5p/HMGA2	CDH1	VIM, CDH2
Prostate cancer (114)	Downregulation	miR-219-5p/HMGA2	CDH1	VIM, CDH2
Prostate cancer (115)	Downregulation	miR-154/HMGA2	CDH1	VIM
Oral squamous cell carcinoma (116)	Downregulation	miR-150/HMGA2	CDH1	VIM, CDH2
Human cervical cancer (117)	Downregulation	miR-142-3p/HMGA2	CDH1	VIM, CDH2
Nasopharyngeal carcinoma (118)	Downregulation	let-7a/HMGA2	CDH1	VIM, Snail, Slug
Head and neck squamous cell carcinoma (119)	Upregulation	hsa_circ_0000264/hsa-let-7b-5p/HMGA2	VIM, Snail, Slug	CDH1
Bladder cancer (120)	Upregulation	circ_0000658/miR-498/HMGA2	CDH2, Slug, Snail, ZEB1, TWIST1	CDH1
Hepatocellular carcinoma (19)	Upregulation	circHPS5/HMGA2	Slug, Snail, VIM	CDH1
Nasopharyngeal carcinoma (121)	Upregulation	HOXC13-AS/miR-383-3p/HMGA2	VIM	CDH1
Bladder cancer (122)	Upregulation	lncRNA LINC00355/miR-424-5p/HMGA2	VIM, ZEB1	CDH1
Hepatocellular carcinoma (69)	Upregulation	SNHG16/let-7b-5p/HMGA2	Slug, CDH2, VIM	CDH1, CTNNA
Oral tongue squamous cell carcinoma (123)	Upregulation	lncRNA HOTTIP/HMGA2/WNT/β-catenin	β-Catenin, c-Myc	CDH1
Tongue squamous cell carcinoma (124)	Upregulation	H19/miR-let-7/HMGA2	TWIST1, ZEB1, Snail	CDH1
Osteosarcoma (122)	Upregulation	LINC01116/miR-424-5p/HMGA2	VIM, CDH2	CDH1

BACH1, BTB domain and CNC homolog 1; CDH1, cadherin 1; CDH2, cadherin 2; CTNNA, alpha-catenin; β-catenin, beta-catenin; EMT, epithelial–mesenchymal transition; ERK, extracellular signal-regulated kinase; H19, H19 imprinted maternally expressed transcript; HMGA2, high mobility group AT-hook 2; HOTTIP, HOXA distal transcript antisense RNA; HOXC13-AS, HOXC13 antisense RNA; HPS5, HPS5 biogenesis of lysosomal organelles complex 2 subunit 2; LINC, long intergenic non-protein coding RNA; lncRNA, long non-coding RNA; MAPK, mitogen-activated protein kinase; N/A, not available; POSTN, periostin; SNHG16, small nucleolar RNA host gene 16; TGFB, transforming growth factor beta; TWIST1, twist family bHLH transcription factor 1; VIM, vimentin; ZEB1, zinc finger E-box-binding homeobox 1.

## 4 Gene therapy methods

### 4.1 Short-interfering RNA

RNA interference (RNAi) is among the most commonly used and important gene therapy methods, involving the use of short-interfering RNAs (siRNAs) (130). The term siRNA refers to a sequence specifically designed to silence the expression of a target gene. Such sequences are currently used in cancer research (*in-vitro* cells or *in-vivo* animal models), providing promising options for the targeted treatment of cancer and other diseases (131, 132). Research demonstrated that the transfection of cells with HMGA2 siRNA markedly suppressed HMGA2 expression, reduced the levels of EMT-related genes, and alleviated the migratory capacity of A549 cells (131). In ACHN cells, the expression of CDH1 was upregulated, whereas that of CDH2 and Snail was downregulated in the tumors treated with HMGA2 shRNA. These results implied that HMGA2 shRNA may be a treatment strategy for renal cell carcinoma (108). In recent years, nanoparticles have attracted attention due to their application in RNAi. The selection of nanoparticles for the molecular delivery of RNAi is mainly attributed to their unique advantages over other carriers (130). Eivazy et al. delivered HMGA2 siRNA by trimethyl chitosan nanoparticles. The delivery of HMGA2 siRNA significantly reduced the expression of HMGA2 and VIM, whereas it increased that of CDH1 (133).

### 4.2 Clustered regular interval short palindromic repeat sequences/CRISPR-associated protein 9 RNA

Clustered regular interval short palindromic repeat sequences/CRISPR-associated protein 9 (CRISPR/Cas9) RNA nucleases are a powerful reverse-genetic tool that can easily achieve targeted editing of multiple genes, thereby inducing complete gene knockout (134). The use of the CRISPR/Cas9 system for targeted gene therapy against tumors has been widely reported. In doxorubicin-resistant BC cells, flow cytometric analysis showed that targeting MDR1 using the CRISPR/Cas9 system increased drug accumulation within the cell compared with untreated cells (135). Song et al. found an indirect increase in the expression levels of HMGA2 and SRY-box transcription factor 9 (SOX9) after CRISPR/Cas9-based knockout of neurofibromin 1 (NF1; a tumor suppressor mutated in neurofibromatosis). These data suggested that NF1 plays a key role as a liver tumor suppressor by negatively regulating HMGA2 and that NF1 and HMGA2 may be useful prognostic or therapeutic indicators (136). In addition, in papillary thyroid carcinoma cells, the CRISPR/Cas9-mediated knockout of HMGA2 inhibited cell proliferation and invasion. It was suggested that HMGA2 knockout blocked the cell cycle in the G2/M phase and promoted cell necrosis (135). The results mentioned above showed that targeting HMGA2 using CRISPR/Cas9 technology can reduce drug resistance in cancers. Using the CRISPR/Cas9 technology and targeting HMGA2 could inhibit the progression of cancer. However, further investigation should be conducted in various types of cancer. Furthermore, the CRISPR/Cas9 technique has limitations, including safe and efficient cell delivery, off-target mutagenesis, and potential immunogenicity. Hence, effective solutions are required to overcome the limitations of this technique (127, 137, 138).

### 4.3 Proteolysis-targeting chimeras

At present, proteolysis-targeting chimeras (PROTACs) have been developed as a useful technology for targeted protein degradation (139). Designed hydrophobic tagging (HyT) probes are synthesized by covalently connecting the hydrophobic portion to the ligand of target nuclear proteins [protein of interest (POI)] (140, 141). The binary POI–HyT complex can simulate the partial denaturation state of protein degradation, and the most commonly used hydrophobic parts include adamantane and tert-butyl carbamate (BOC_3_) arginine (141). PROTACs can induce the dynamic degradation of intracellular proteins or POIs. Thus, they play an important role in addressing drug resistance by degrading the pathogenic protein without compensatory increase or mutation (142). Unlike nucleic acid-based techniques for protein regulation, such as RNAi and CRISPR/Cas9, these low immunogenicity chimeras cause reversible and rapid target depletion (143). In addition, PROTACs can be recovered after POI ubiquitination and degradation, allowing these molecules to recatalyze the elimination of additional POIs (143). Thus far, the treatment strategy involving the use of PROTACs has been successfully applied to conditionally degrade approximately 50 proteins *in vitro* and *in vivo*, including bromodomain containing 4-targeting (BRD4-targeting) PROTACs, cereblon-based (CRBN-based) PROTACs, MCL1 apoptosis regulator, BCL2 family member-based (MCL1-based) PROTACs, and STAT3-based PROTACs (144). For example, Wang et al. developed the efficient STAT3 inhibitor SI-109 and used it to develop PROTAC SD-36 targeting STAT3. The results showed that, at low nanomolar concentrations, SD-36 effectively reduces STAT3 in numerous types of leukemia and lymphoma cells (145). Furthermore, Crews et al. synthesized the first PROTAC DAS-2-2-6-CRBN targeting BCR-ABL. This PROTAC resulted in efficient BCR-ABL degradation and growth inhibition in chronic myeloid leukemia K562 cells (146). Research has revealed the presence of >600 E3 ubiquitin ligases in humans; many of those can be used to design PROTACs (147). Thus, further research studies are warranted to identify alternative therapies based on PROTAC-mediated degradation of HMGA2.

## 5 Conclusions and perspectives

HMGA2 is overexpressed in multiple types of cancer and has been associated with the EMT process and tumor invasion. Thus, targeting HMGA2 may provide multiple benefits in terms of tumor growth, the EMT phenotype, metastasis, and invasion. These effects indicate that HMGA2 is a promising target for enhancing cancer therapy and improving the patient survival rate. Most recent studies have demonstrated that HMGA2 is highly expressed in cancer and linked to the EMT, invasion, and poor prognosis. HMGA2 acts as a key factor in the complex networks of the TGFβ, MAPK, and WNT/β-catenin signaling pathways involved in the EMT process and invasion of tumor cells. Furthermore, most non-coding RNAs (miRNAs, lncRNAs, and circRNAs) participate in the regulation of HMGA2 expression in cancer to affect EMT. In addition, evidence has indicated that HMGA2 siRNA and CRISPR/Cas9-mediated knockout of HMGA2 serve as potential therapeutic approaches by suppressing HMGA2 for the treatment of cancer. However, currently, there are limited treatment options targeting the inhibition of HMGA2 expression to mitigate EMT and invasion of cancer. For example, small molecule inhibitors targeting HMGA2 have not yet been studied or identified. Thus far, there is a lack of drugs targeting HMGA2 to delay EMT and invasion of cancer. Gene modification strategies (e.g., acetylation, methylation, and ubiquitination) targeting HMGA2 are urgently required for the treatment of cancer. This approach may suppress EMT and increase the survival rate of patients with cancer. Furthermore, it is necessary to elucidate the specific molecular mechanism through which HMGA2 mediates the EMT process in cancer. Such knowledge will contribute to the discovery of more effective treatment strategies for inhibiting tumor metastasis and controlling resistance to chemotherapy.

In conclusion, targeting HMGA2 through direct and indirect regulation offers a promising direction for antitumor therapy.

## Author contributions

QM: Formal Analysis, Writing – original draft. SY: Writing – original draft, Formal Analysis. HL: Conceptualization, Investigation, Writing – review & editing. YZ: Conceptualization, Writing – review & editing, Supervision. YM: Conceptualization, Writing – review & editing, Investigation. WZ: Writing – review & editing, Writing – original draft.
